# Reorganization of motor network in patients with Parkinson's disease after deep brain stimulation

**DOI:** 10.1111/cns.14792

**Published:** 2024-06-12

**Authors:** Bei Luo, Lei Chang, Chang Qiu, Wenwen Dong, Liang Zhao, Yue Lu, Jian Sun, Jiuqi Yan, Xiang Wei, Jun Yan, Wenbin Zhang

**Affiliations:** ^1^ Department of Functional Neurosurgery, Affiliated Nanjing Brain Hospital Nanjing Medical University Nanjing China; ^2^ Department of Geriatric Neurology, Affiliated Nanjing Brain Hospital Nanjing Medical University Nanjing China

**Keywords:** deep brain stimulation, functional connectivity, microlesion effect, Parkinson's disease, resting‐state functional magnetic resonance

## Abstract

**Aims:**

Parkinson's disease (PD) patients experience improvement in motor symptoms after deep brain stimulation (DBS) and before initiating stimulation. This is called the microlesion effect. However, the mechanism remains unclear. The study aims to comprehensively explore the changes in functional connectivity (FC) patterns in movement‐related brain regions in PD patients during the microlesion phase through seed‐based FC analysis.

**Methods:**

The study collected the resting functional magnetic resonance imaging data of 49 PD patients before and after DBS surgery (off stimulation). The cortical and subcortical areas related to motor function were selected for seed‐based FC analysis. Meanwhile, their relationship with the motor scale was investigated.

**Results:**

The motor‐related brain regions were selected as the seed point, and we observed various FC declines within the motor network brain regions. These declines were primarily in the left middle temporal gyrus, bilateral middle frontal gyrus, right supplementary motor area, left precentral gyrus, left postcentral gyrus, left inferior frontal gyrus, and right superior frontal gyrus after DBS.

**Conclusion:**

The movement‐related network was extensively reorganized during the microlesion period. The study provided new information on enhancing motor function from the network level post‐DBS.

## Graphical abstract

Seed‐based functional connectivity analysis revealed significant alterations in motor‐related networks post‐DBS surgery. A significant and widespread reduction in FC in motor‐related network brain regions, particularly within the left middle temporal gyrus, bilateral middle frontal gyrus, right supplementary motor area, left precentral gyrus, and left postcentral gyrus after DBS.

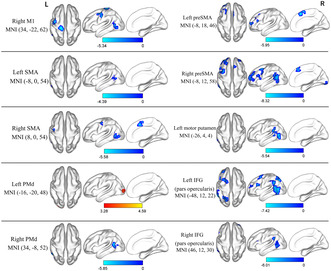

## 1 INTRODUCTION

Subthalamic nucleus deep brain stimulation (STN‐DBS) is the primary surgical treatment for Parkinson's disease (PD).^1^, ^2^, ^3^ After DBS, PD patients often wait 2–3 weeks before starting electrical stimulation. During this period, the symptoms of PD patients improved.^4^ This phenomenon is called the microlesion effect (MLE)^5^, ^6^ and lasts several days to weeks. However, the mechanisms behind MLE require elucidation. Some studies suggest that MLE was related to the abnormal basal ganglia output induced by cell and fiber destruction in the target nucleus. In addition, MLE may be linked with local destruction of implanted electrodes. This leads to injury and subsequent peripheral neuronal or glial cell loss, the release of neurotransmitters from local neuron destruction, and edema around the electrode locus due to injury.^7^, ^8^ MLE occurrence was considered a sign of good placement of the DBS electrode inside a specific part of the target structure.^9^, ^10^ MLE may be an effective STN‐DBS predictor in treating PD.^11^, ^12^ Therefore, investigating the MLE mechanism is essential, as it could be conducive to deciphering the DBS mechanism.

Research suggests that DBS improves symptoms by modulating brain networks with electrical stimulation.^13^ Thus, functional magnetic resonance imaging (fMRI) may be an excellent way to explore the MLE mechanism. Only a few studies have utilized fMRI to investigate the correlation between motor symptoms and neuroimaging abnormalities in MLE. Jech et al.^14^ observed the activation of relevant brain regions postoperatively lower in the bilateral primary sensorimotor cortex, thalamus postcentral, and superior temporal gyri than preoperatively within the tapping experiment. However, the effect of the task itself on brain function questions the result. Holiga et al.^8^ used the eigenvector centrality method to analyze pre‐ and post‐operative fMRI data of 13 PD patients. Electrode penetration was linked with increased eigenvector centrality in brain stem functional connections. However, the sample size limited the result accuracy of the results.^8^ Our previous work has demonstrated alterations in brain activation in motor‐related brain areas, such as the precentral gyrus and supplementary motor area (SMA), during microlesion after DBS.^15^, ^16^ However, they are yet to explore the change in the motor network.

Therefore, we obtained resting‐state fMRI data of 49 PD patients before and after DBS surgery and selected cortical and subcortical regions of interest (ROIs) associated with motor function for seed‐based functional connectivity (FC) analysis. This would help understand the motor network changes during the microlesion period. Finally, the correlation between resting‐state FC and clinical scores of motor symptoms was tested for each seed.

## 2 MATERIALS AND METHODS

### 2.1 Participants and scales

Initially, 57 patients were enrolled in the study. Based on the United Kingdom Parkinson's Disease Society Brain Bank clinical diagnostic criteria,^17^ the PD patients were jointly diagnosed by experienced neurologists and neurosurgeons after satisfying all the DBS surgery indications. Exclusion criteria for PD patients: other central nervous system diseases, taking drugs affecting brain function, and inability to tolerate MRI scans. All the study data were obtained from the Brain Hospital affiliated with Nanjing Medical University and approved by the ethics committee of the hospital. All the patients signed informed consent forms before the study. The cognitive and mental states of all participants were assessed using the Montreal Cognitive Assessment (MoCA), Hamilton Anxiety Rating Scale (HAMA), and Hamilton Depression Rating Scale (HAMD). We used the Unified Parkinson's Disease Rating Scale part‐III (UPDRS‐III) to determine motor symptoms in PD patients 3 days before (24–72 h before DBS) and 1 day after (24–48 h after DBS surgery) DBS. All data were collected with the stimulus turned off. Data were obtained at least 12 h after discontinuation of antiparkinsonian drugs to reduce the drug effect on the experiment.

### 2.2 Surgery

All the PD patients underwent standard stereotactic frame surgery by a single surgeon. Bilateral STN was identified as a DBS target. Preoperative brain 3.0T MRI and skull CT with frame data were imported within the operation planning software to devise an operation plan. The midpoint of the anterior and posterior commissures became the origin of creating a coordinate system. The STN target was 11–12 mm next to the origin, 3 mm back, and 4 mm down. Moreover, the target coordinates were fine‐tuned according to the specific STN nucleus location within the MRI image. The surgery was divided into two phases. The first stage was implanting the intracerebral electrode (model L301, PINS, China) under local anesthesia, and the second was placing the pulse emitter (model G102R, PINS) under general anesthesia.

### 2.3 Image acquisition

MRI data were scanned using a 1.5T MRI scanner (GE Medical System) with an 8‐channel head coil. The resting‐state fMRI data were acquired with an echo planar imaging (EPI) sequence: echo time (TE) = 40 ms, repetition time (TR) = 2000 ms, flip angle (FA) = 90°, matrix size = 64 × 64, field of view (FOV) = 240 × 240 mm, number of slices = 28, thickness = 3.0 mm with no gap, voxel size = 3.75 × 3.75 × 3 mm^3^, and number of total volumes = 128. Three‐dimensional T1‐weighted images were obtained using a 3D magnetization‐prepared rapid gradient‐echo (MPRAGE) sequence with the following parameters: TE = 4.932 ms, TR = 11.864 ms, FA = 20°, matrix size = 256 × 256, FOV = 152 × 152 mm, number of slices = 112, thickness = 1.4 mm, and voxel size = 0.59 × 0.59 × 1.4 mm^3^. All the heads of the participants were secured using foam pads, and earplugs helped reduce noise during the scan. Each patient was asked to close their eyes, relax, and not think about anything during the scan.

### 2.4 Data preprocessing

Structural and functional images were processed with resting‐state fMRI data processing assistant (DPABI_7.0, http://rfmri.org/DPABI)^18^ using MATLAB2013b (http://www.mathworks.com/products/matlab/). The preprocessing steps were summarized accordingly: (1) The first five time points were discarded to rule out the initial signal instability; (2) slice‐timing correction and head motion correction were performed. PD patients possessing excessive head movement (head translation or rotation >3.0 mm or 3.0°) were excluded; (3) the remaining images were normalized to the Montreal Neurological Institute (MNI) template and resampled to 3 × 3 × 3 mm^3^; (4) the covariates of 24 movement parameters, white matter signals, and cerebrospinal fluid signals were removed via regression; (5) the data were spatially smoothed using a 6‐mm full width at half‐maximum Gaussian kernel and detrending and bandpass filtering (0.01–0.10 Hz). Eight patients were excluded because of excessive head movement.

### 2.5 Seed FC analysis

Cortical and subcortical seeds associated with motor function as ROIs involved the bilateral primary motor cortex (M1), bilateral SMA, bilateral dorsolateral premotor cortex (PMd), bilateral pre‐SMA, bilateral motor putamen, and pars opercularis of the bilateral inferior frontal gyrus (IFG). All the chosen seeds are the higher motor cortex, associated with motor initiation, execution, control, or coordination. The peak seed coordinates were obtained from activation likelihood estimation from previous studies.^19^, ^20^, ^21^, ^22^, ^23^, ^24^, ^25^ The seeds were defined as spherical ROIs with a 6 mm radius around the coordinates. Seed‐based FC analysis was performed voxel‐wise on the REST Toolkit (http://www.restfmri.net). First, we extracted the average bold‐signal time course in each ROI and tested its correlation with every other voxel in the brain using a bivariate Pearson's correlation. Finally, the Fisher r–z helped transform the results for further statistical analysis.

### 2.6 Statistical analysis

Repeated measures of analysis of variance helped determine the differences in UPDRS‐III scores of patients at different time points with the SPSS 22.0 software (IBM Corp.). A paired *t*‐test helped detect differences in whole‐brain FC at each seed in PD patients before and after DBS surgery, with the mean frame displacement value as a covariate. These thresholds were applied in the results: uncorrected threshold *p*‐value < 0.001 and family wise error rate‐corrected cluster extent threshold *p*‐value < 0.05.

### 2.7 Correlation analysis

The study determined whether a correlation existed between FC and clinical motor scales. The FC brain area values that differed before and after surgery were extracted for each seed, and their correlation using the preoperative UPDRS‐III score was measured. Moreover, we tested whether altered FC values before and after DBS surgery were associated with a decline in UPDRS‐III scores.

## 3 RESULTS

### 3.1 Demographic and clinical characteristics

The clinical characteristics of PD patients are represented in Table 1. One day post‐surgery, the UPDRSIII score of PD patients decreased to 30.39 ± 9.34 from 39.73 ± 12.56 points before surgery (*p* < 0.001). The UPDRS‐III returned to preoperative levels after 1 month of surgery (*p* = 0.872). These results depicted the transient MLE character.

**TABLE 1:** Demographic and clinical data of all subjects.

	PD (*n* = 49) Mean ± SD	*p*‐Value
Age (years)	61.45 ± 9.38	–
Sex (male/female)	22 ± 27	–
HAMA	6.27 ± 3.72	–
HAMD	6.59 ± 4.03	–
MoCA	23.51 ± 5.42	
UPDRS‐III		
Three days before DBS	39.73 ± 12.56	<0.001^a^, *
One day after DBS	30.39 ± 9.34	–
One month after DBS	40.41 ± 12.02	–

Abbreviations: DBS, deep brain stimulation; HAMA, Hamilton Anxiety; HAMD, Hamilton Depression; Mean ± SD, mean ± standard deviation; MoCA, Montreal Cognitive Assessment; PD, Parkinson's disease; UPDRSD‐III, Unified Parkinson's Disease Rating Scale part‐III.

^a^ Repeated measures ANOVA test.

* *p* < 0.05.

### 3.2 Seed‐based FC analysis

#### 3.2.1 FC of the M1 in the resting state

**FIGURE 1:**
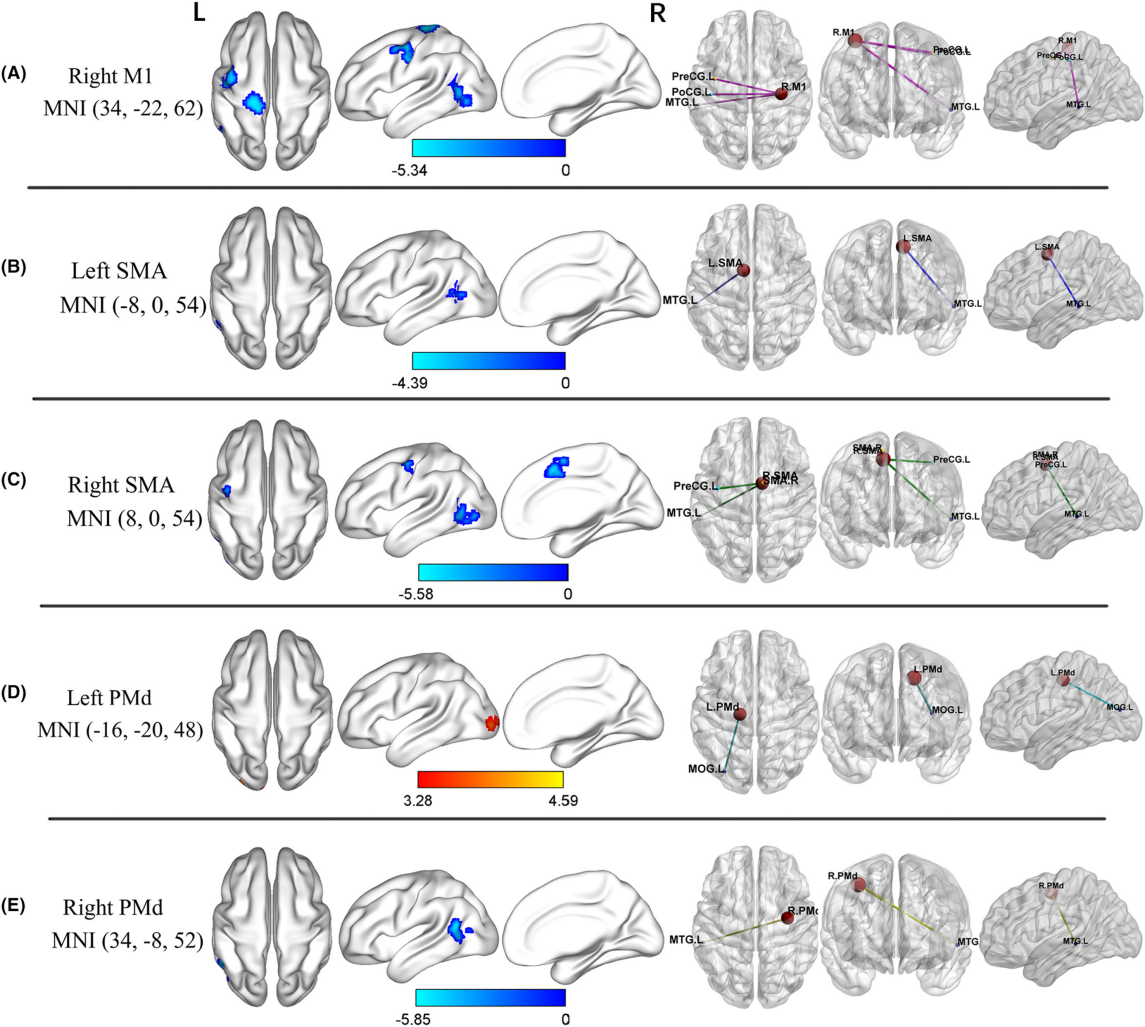
Brain regions with altered FC in PD patients before and after DBS surgery (voxel *p* < 0.001, FWE correction with cluster *p* < 0.05). Right M1 (A), left SMA (B), right SMA (C), left PMd (D), and right PMd (E) for seed‐based FC analysis; L, left and R, right hemispheres; M1, primary motor cortex; PMd, dorsolateral premotor cortex; SMA, supplementary motor area.

With the right M1 as the seed point, PD patients exhibited reduced FC in the left middle temporal gyrus, left postcentral gyrus, and left precentral gyrus post‐DBS surgery (Table 2, Figure 1A). No significant results could be observed when the left M1 was chosen as seed.

**TABLE 2:** Alterations of FC in PD patients before and after DBS surgery.

Seed area	Brain region (AAL)	Cluster size	Peak MNI coordinate			Peak intensity
PD‐Pre‐DBS > PD‐Post‐DBS						
Right M1						
Cluster 1	Temporal_Mid_L	154	−54	−63	9	−4.7679
Cluster 2	Precentral_L	128	−45	−9	39	−5.2407
Cluster 3	Postcentral_L	78	−18	−30	72	−5.3357
Left SMA						
Cluster 1	Temporal_Mid_L	90	−54	−63	9	−4.3855
Right SMA						
Cluster 1	Temporal_Mid_L	155	−54	−72	3	−5.5847
Cluster 2	Precentral_L	51	−33	−18	42	−5.1131
Cluster 3	Supp_Motor_Area_R	91	0	9	54	−5.0401
Right PMd						
Cluster 1	Temporal_Mid_L	153	−54	−57	18	−5.8505
Left pre‐SMA						
Cluster 1	Frontal_Mid_L	67	−30	57	9	−5.9504
Cluster 2	Frontal_Mid_L	68	−27	30	33	−4.9268
Cluster 3	Supp_Motor_Area_R	89	6	21	51	−5.1965
Right pre‐SMA						
Cluster 1	Lingual_R	66	15	−90	−9	−5.4394
Cluster 2	Temporal_Mid_L	393	−57	−54	18	−8.3236
Cluster 3	Frontal_Mid_R	96	30	57	18	−5.6321
Cluster 4	Frontal_Mid_L	263	−30	51	9	−6.4204
Cluster 5	Frontal_Inf_Tri_L	66	−42	21	21	−4.7072
Cluster 6	Frontal_Mid_L	123	−39	6	51	−5.1084
Cluster 7	Frontal_Sup_Medial_R	79	9	27	51	−5.3351
Left motor putamen						
Cluster 1	Temporal_Mid_L	69	−51	−66	6	−4.949
Cluster 2	Temporal_Mid_L/SupraMarginal_L	109	−60	−45	6	−5.5405
Left IFG (pars opercularis)						
Cluster 1	Temporal_Mid_L	599	−51	−63	9	−7.4204
Cluster 2	Frontal_Mid_L	60	−30	57	18	−4.9474
Cluster 3	Frontal_Inf_Tri_L	64	−42	21	27	−4.8937
Cluster 4	Parietal_Sup_L	161	−27	−66	45	−4.9943
Cluster 5	Frontal_Mid_L	98	−33	15	51	−4.4436
Right IFG (pars opercularis)						
Cluster 1	Temporal_Mid_L	277	−60	−60	6	−6.0064
Cluster 2	Frontal_Sup_R	54	33	57	9	−5.002
Cluster 3	Frontal_Inf_Tri_L	55	−60	15	21	−4.7051
PD‐Post‐DBS > PD‐Pre‐DBS						
Left PMd						
Cluster 1	Occipital_Mid_L	75	−33	−93	0	4.5862

Abbreviations: AAL, anatomical automatic labeling; FC, functional connectivity; IFG, inferior frontal gyrus; M1, primary motor cortex; MNI, Montreal Neurological Institute; PD‐Post‐DBS, 1 day after DBS; PD‐Pre‐DBS, before DBS; PMd, dorsolateral premotor cortex; SMA, supplementary motor area.

#### 3.2.2 FC of the SMA in the resting state

Parkinson's disease patients displayed similar reduced FC in the left middle temporal gyrus after DBS, with the left and right SMA as seed regions (Table 2, Figure 1B,C). Additionally, the right SMA revealed lower FC within the left precentral gyrus.

#### 3.2.3 FC of the PMd in the resting state

A significantly increased left PMd FC was observed in the left middle occipital gyrus (Table 2, Figure 1D). However, PD patients had decreased FC in the left middle temporal gyrus for the right PMd seed (Table 2, Figure 1E).

#### 3.2.4 FC of the pre‐SMA in the resting state

**FIGURE 2:**
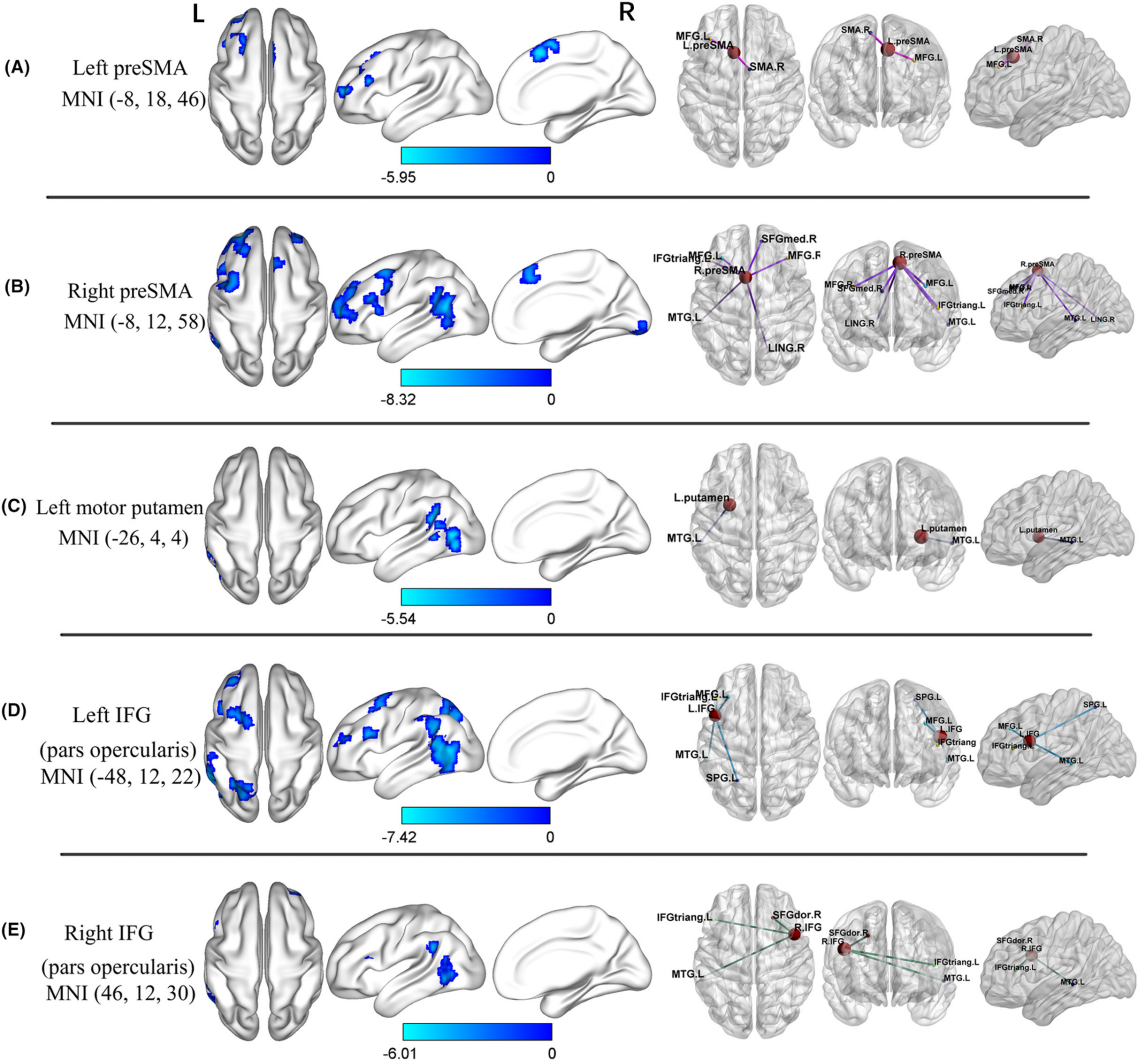
Brain regions with altered FC in PD patients before and after DBS surgery (voxel *p* < 0.001, FWE correction with cluster *p* < 0.05). Left pre‐SMA (A), right pre‐SMA (B), left motor putamen (C), left IFG (pars opercularis) (D), right IFG (pars opercularis) (E) for seed‐based FC analysis; IFG, inferior frontal gyrus; SMA, supplementary motor area.

We observed decreased FC between the left pre‐SMA seed, the left middle frontal gyrus, and the right SMA (Table 2, Figure 2A). With the right pre‐SMA as the seed point, PD patients had reduced FC in the right lingual gyrus, left middle temporal gyrus, left IFG (triangular part), right superior frontal gyrus (medial), and bilateral middle frontal gyrus (Table 2, Figure 2B).

#### 3.2.5 FC of the motor putamen in the resting state

After DBS, the left motor putamen FC was significantly decreased with the left middle temporal gyrus and left supramarginal gyrus (Table 2, Figure 2C). Nevertheless, no altered FC values could be observed in the right motor putamen.

#### 3.2.6 FC of the IFG (pars opercularis) in the resting state

Parkinson's disease patients had reduced FC in the left middle temporal gyrus, left middle frontal gyrus, left superior parietal gyrus, and left IFG (triangular part), with the left IFG (pars opercularis) as the seed point (Table 2, Figure 2D). Meanwhile, PD patients had reduced FC in the left middle temporal gyrus, right superior frontal gyrus (dorsolateral), and left IFG (triangular part) with the seed point being the right IFG (pars opercularis) (Table 2, Figure 2E).

### 3.3 Correlation analysis

**FIGURE 3:**
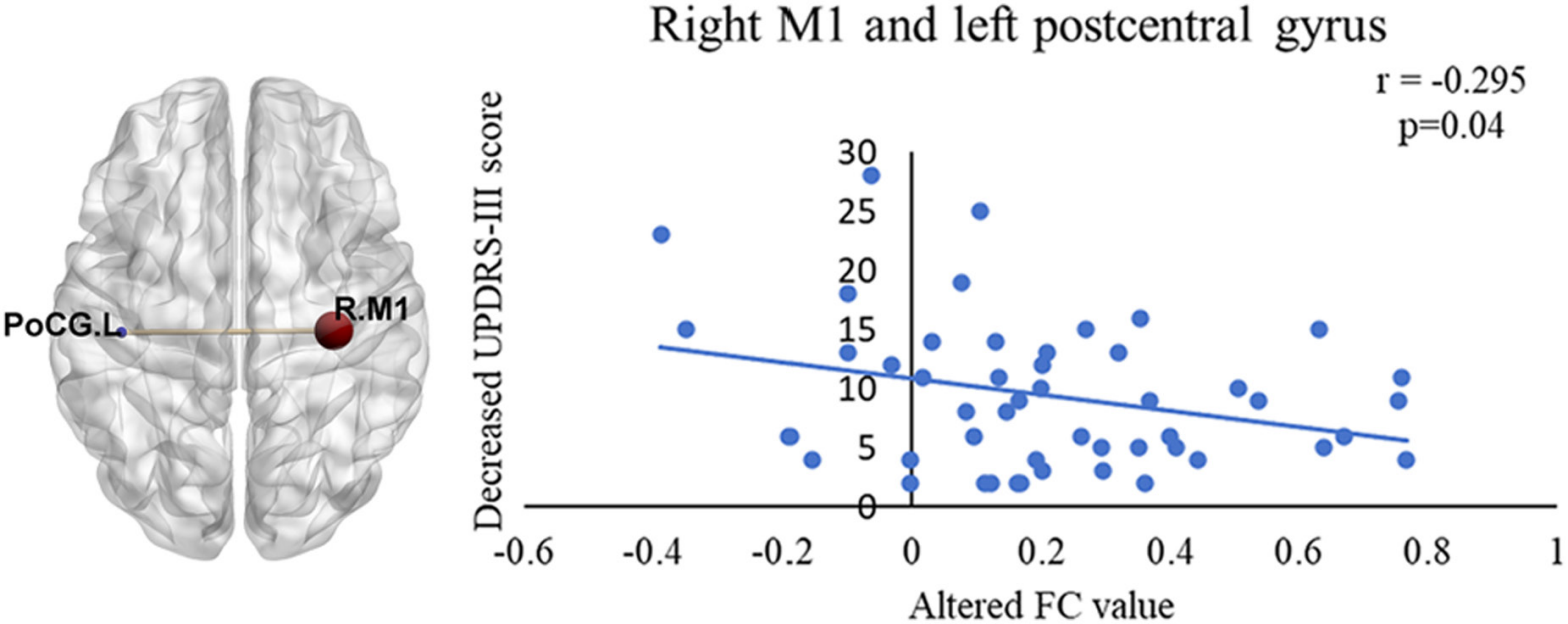
The correlation between decreased UPDRS‐III and altered FC between the right M1 and left postcentral gyrus. FC, functional connectivity; M1, primary motor cortex; UPDRS‐III, Unified Parkinson's Disease Rating Scale part‐III.

The altered FC value between the left postcentral gyrus and right M1 was negatively related to reduced UPDRS‐III scores before and after surgery (Figure 3).

## 4 DISCUSSION

The current study was a comprehensive FC analysis using cortical and subcortical motor‐related regions as seeds to decipher the changes in motor networks during MLE. During the study implementation, the same physician team performed all the target planning and surgical operations. Hence, despite the implant target of each patient being slightly different, the study results were unaffected. The study results revealed significant alterations in motor‐related networks post‐DBS surgery. A significant and widespread reduction in FC in motor‐related network brain regions, particularly in the left middle temporal gyrus, bilateral middle frontal gyrus, right SMA, left precentral gyrus, and left postcentral gyrus after DBS. Interestingly, the FC values between the left PMd FC and left middle occipital gyrus were enhanced. Moreover, the negative correlation between altered right M1 FC with the left postcentral gyrus and decreased UPDRS‐III score developed the relationship between the motor network and enhanced PD motor function.

This study observed that the connections between M1 and brain regions in the sensorimotor network were significantly reduced after DBS. M1 is a critical brain region for motor planning and execution.^26^, ^27^ Striatal dopamine depletion induces changes in neural activity within the cortico‐basal ganglia motor loop, leading to motor output dysfunction. A key motor output regulator is the balance between excitation and inhibition in the M1. Cortical disinhibition could be a prodromal feature of PD.^28^ Continuous movement helped activate the M1 and primary somatosensory cortex.^29^ Multiple comparative brain function studies in PD patients and healthy controls (HC) based on various motor tasks have identified overactivated M1 and PMd.^30^, ^31^, ^32^, ^33^, ^34^ Haslinger et al.^33^ identified that PD patients had elevated bilateral M1 activity before and after levodopa compared to HC. After levodopa treatment, M1 activation was reduced in PD patients, and levodopa could relatively normalize hyperactivation with impaired motor cortex function.^33^ In a PET study on PD, levodopa could significantly decrease regional glucose metabolism in the left M1, and the degree of network inhibition was linked with clinical improvement.^35^ Therefore, the FC decoupling of M1 induced by implanting electrodes could normalize M1 function during the microlesion period after DBS, improving motor symptoms. The reduction in FC between the right PMd and left middle temporal gyrus may be involved in the above process.

SMA is part of the motor system of the prefrontal cortex and is associated with complex motor preparation and control.^36^ SMA is divided into pre‐SMA and caudal SMA proper.^37^ SMA has dense projections on the spinal cord and brain stem reticular structures and is linked with motor initiation.^37^, ^38^ A common finding among various imaging studies investigating brain activity during self‐initiation was SMA hypoactivation in PD patients compared to normals.^30^, ^39^, ^40^, ^41^ Wu et al.^41^ observed that PD patients had decreased regional homogeneity (ReHo) in the SMA compared to healthy controls. In a recent study, the ReHo of SMA was elevated during MLE after DBS surgery,^16^ suggesting that DBS relatively normalized SMA activity. The prefrontal cortex helps plan, regulate, and control psychological activities. The prefrontal cortex is essential in advanced and purposeful behaviors.^42^, ^43^, ^44^ Moraschi et al.^45^ analyzed task‐state cortical activation areas in six PD patients with early stage hemiparkinsonian. They observed that the dorsolateral prefrontal cortex of the affected hemisphere indicated more robust activation than the healthy hemisphere. Frontal lobe activation is more prevalent during verbal fluency (VF) tasks.^46^ Ci et al.^47^ observed that the VF task primarily activated the left middle frontal gyrus and left superior frontal gyrus while studying the positioning of Chinese functional areas in the frontal and temporal lobes using the Mandarin task paradigm. A positive correlation was observed between striatal and frontal dopaminergic activity and cognitive function inside the executive and language domains in a group of early onset PD patients.^46^ The middle frontal gyrus was comparable to Broca's area based on its ability to identify linguistic hemispheric dominance through resting‐state fMRI.^48^ The present study showed a widespread decline in FC between the seed point of motor preparation regions and many prefrontal and temporal lobe areas post‐DBS. SMA decoupling from motor‐related areas may decrease compensatory activity in related brain areas.

The pathological feature of PD is losing dopaminergic neurons within the substantia nigra. Neuronal degeneration of the substantia nigra affects the ventrolateral cell population, which projects posterolaterally to the putamen forming Lewy bodies comprising aggregated α‐synuclein.^49^ The putamen was linked while planning the self‐initiated or self‐paced movements.^34^ Several studies have established decreased putamen activity in PD patients.^41^, ^50^ The middle temporal gyrus was linked with cognitive function,^51^ especially VF.^52^ Many studies have indicated that cognitive function, particularly speech fluency, was significantly decreased after DBS.^53^, ^54^, ^55^ The FC decline between the putamen and left middle temporal gyrus could depict a reduction in cognitive function post‐DBS. The left IFG possesses the traditional Broca's area and is integral to finishing language production in humans. It is associated with all language function aspects, such as speech production and memory.^56^, ^57^ The pars opercularis of the left IFG is the primary hub within the language network and is associated with many brain regions.^58^ The study observed a modest positive association between the degree of IFG activation in children and the absolute number of words synthesized during language assessment.^59^ Therefore, reduced cognitive function after DBS could be related to the decline in IFG FC with the frontal and temporal lobes due to electrode implantation.

The study has several limitations. First, antiparkinsonian drugs were discontinued for more than 12 h before data collection. However, levodopa accumulation may affect brain function. Second, the field strength involved in the study was 1.5T, with a short scanning time. Therefore, 3.0T and extended MRI acquisition time can help verify the research results. Finally, follow‐up collections can help explore ongoing brain function changes.

## 5 CONCLUSIONS

In this study, the changes in the motor network during the microlesion period post‐DBS were deciphered using seed‐based FC analysis. After DBS, the motor network was reorganized using extensive FC reduction. Therefore, our findings provide a greater emphasis on motor networks when studying DBS.

## AUTHOR CONTRIBUTIONS

BL and LC contributed equally to this work. Data collection and analysis were performed by BL, LC, JS, YL, and WD. Statistical analysis were performed by BL, LC, CQ, JY, XW, and LZ. The first draft of the manuscript was written by BL, LC, and CQ. WZ and JY edited and revised the manuscript. All authors contributed to and approved the final manuscript.

## CONFLICT OF INTEREST STATEMENT

The authors declare that they have no competing interests.

## Data Availability

The data supporting the results of this study can be obtained from the corresponding authors via email.
